# Polymerase Chain Reaction (PCR) as a Potential Point of Care Laboratory Test for Leprosy Diagnosis—A Systematic Review

**DOI:** 10.3390/tropicalmed3040107

**Published:** 2018-10-01

**Authors:** Sushma Tatipally, Aparna Srikantam, Sanjay Kasetty

**Affiliations:** 1 LEPRA Society, Blue Peter Public Health and Research Centre, Cherlapally, Hyderabad 501301, Telangana, India; sushma@leprahealthinaction.in; 2 Formerly at LEPRA Society, Blue Peter Public Health and Research Centre, Cherlapally, Hyderabad 501301, Telangana, India; sanjay.kasetty@gmail.com

**Keywords:** leprosy, leprosy diagnosis, PCR, slit skin smears, point of care test, skin biopsy, early diagnosis

## Abstract

Leprosy is an infectious disease caused by *Mycobacterium leprae* and mainly affects skin, peripheral nerves, and eyes. Suitable tools for providing bacteriological evidence of leprosy are needed for early case detection and appropriate therapeutic management. Ideally these tools are applicable at all health care levels for the effective control of leprosy. This paper presents a systematic review analysis in order to investigate the performance of polymerase chain reaction (PCR) vis-à-vis slit skin smears (SSS) in various clinical settings and its potential usefulness as a routine lab test for leprosy diagnosis. Records of published journal articles were identified through PubMed database search. Twenty-seven articles were included for the analysis. The evidence from this review analysis suggests that PCR on skin biopsy is the ideal diagnostic test. Nevertheless, PCR on SSS samples also seems to be useful with its practical value for application, even at primary care levels. The review findings also indicated the necessity for improving the sensitivity of PCR and further research on specificity in ruling out other clinical conditions that may mimic leprosy. The *M. leprae*-specific repetitive element (RLEP) was the most frequently-used marker although its variable performance across the clinical sites and samples are a matter of concern. Undertaking further research studies with large sample numbers and uniform protocols studied simultaneously across multiple clinical sites is recommended to address these issues.

## 1. Introduction

Leprosy is an infectious disease caused by *Mycobacterium leprae* and mainly affects skin, peripheral nerves, and eyes [1,2,3]. Leprosy has long-term consequences on the structure and function of the peripheral nerves leading to disabilities in limbs, which impact on the socioeconomic well-being of the affected individuals [4]. Despite three decades of effective treatment with multidrug therapy (MDT), leprosy persists as a public health problem in many regions of the world [5]. Each year 300,000 people are newly diagnosed with leprosy worldwide; half are reported from India [6]. Despite the World Health Organization (WHO) declaring the elimination of leprosy as a public health problem in the year 2000, new leprosy cases still continue to occur in India with an annual incidence of around 135,000 cases [7]. There is evidence that many more patients still go undetected due to various reasons including social stigma attached to the disease, which hinders health-care-seeking behavior among affected persons [8]. Hence it is imperative to widen the scope and accuracy of leprosy detection for early identification before the consequences of nerve damage have set in [9]. The current standard diagnosis of leprosy is mostly based on clinical evaluation of patients, except in a few settings where the microscopy of slit skin smears (SSS) for acid-fast bacilli and/or histopathological examination (HPE) of skin biopsies are being used as additional tests [10]. Clinical manifestations of leprosy are determined by patient immune responses to *Mycobacterium leprae*. Leprosy patients are classified by the Ridley–Jopling classification on the basis of the morphology, type, and number of skin lesions, as well as nerve involvement supplemented by the bacterial index (BI) and histopathological examination. The Ridley–Jopling types are tuberculoid (TT), borderline tuberculoid (BT), borderline (BB), borderline lepromatous (BL), lepromatous leprosy (LL), pure neural (PN) and indeterminate (I) [11].

The current operational classification of leprosy used by WHO is based on number of skin lesions; patients with less than five lesions are classified as paucibacillary (PB) and more than five as multibacillary (MB) leprosy. However, this classification, merely based on the number of lesions, may not always hold good for specific treatment strategies, as it has been frequently demonstrated that acid-fast bacilli (AFB) are present in cases clinically classified as PB. Such PB patients with active lesions may potentially be transmitting *M. leprae* to their contacts unless they are treated appropriately [12]. Hence there has been an emphasis on bringing back the laboratory diagnostic component into routine practice [13]. The two traditional tests *viz.* SSS and HPE of biopsies, though still holding well in terms of convenience of usage, have their own inherent limitations. SSS is relatively low in sensitivity and includes the risk of subjective errors of microscopic examination, whereas the HPE has the limitations of long turnaround time and technically demanding laboratory procedures [14]. Hence it is very important to develop diagnostic strategies involving highly sensitive laboratory tests for early detection of leprosy. Suitable tools for providing bacteriological evidence of leprosy are needed for early case detection and appropriate therapeutic management of leprosy. Ideally these tools are applicable at all health care levels for effective control of leprosy.

Molecular diagnosis by nucleic acid amplification test (NAAT) is an emerging science in the clinical management of infectious diseases. Polymerase chain reaction (PCR) is one of the most popular NAAT currently being used for the diagnosis of infectious diseases [15]. Routine clinical use of NAAT has been well established in tuberculosis and other mycobacterial diseases. NAAT has almost replaced the conventional lab diagnostic tests in TB and has become the most widely used test at all levels of health care [16]. Such molecular diagnosis has not yet been practiced in the case of leprosy. PCR nevertheless has been popularly used for drug resistance testing and molecular typing of leprosy but never so far for the routine clinical diagnosis [17,18]. Given the potential use of this very important test, there is a need for a scientific analysis of the effectiveness of PCR for lab diagnosis of leprosy in correlation with the current standards. This evidence is envisaged to help the formulation of better policies for diagnosis and treatment of leprosy. With this background, the present systematic review analysis has been conducted in order to investigate the performance of PCR vis-à-vis SSS in various clinical settings and its potential usefulness as a routine lab test for leprosy diagnosis.

## 2. Materials and Methods

### 2.1. Study Design

The study is a systematic review analysis to assess the usefulness of PCR for the laboratory diagnosis of leprosy. The review has been carried out with the purpose of accruing evidence on the performance of PCR in correlation with clinical classification of leprosy and standard laboratory diagnostic tests. The highlights from the review are envisaged to be useful in addressing the gaps in the existing systems and recommending better strategies for the future application of PCR during routine clinical diagnosis of leprosy. The protocol has been prepared based on Preferred Reporting Items for Systematic reviews and Meta-Analyses (PRISMA) guidelines for systematic review analysis [19].

### 2.2. Data Search

**Figure 1:**
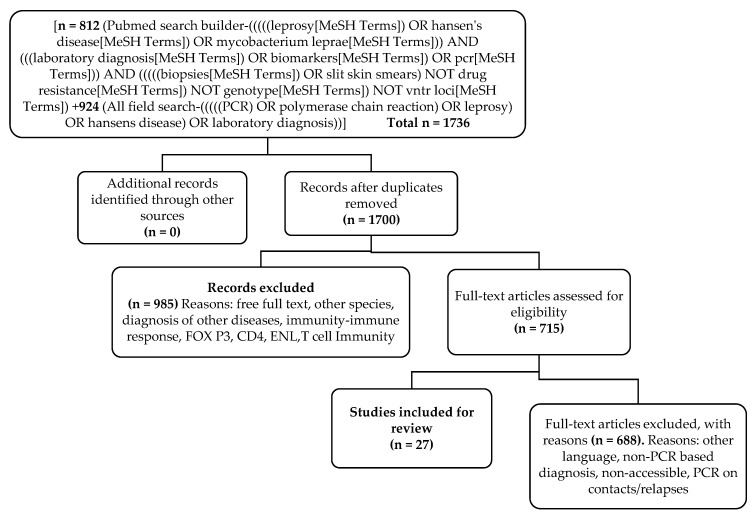
Flow chart detailing review steps (PRISMA guidelines).

Records of published journal articles were identified through searching the PubMed database [20]. Journal articles published until April 2018 were included. The data search was conducted between 15 March 2018 to 4 May 2018. All field searches for PCR and leprosy yielded 924 articles, which were filtered to 812 with Medical Subject Heading (MeSH) terms *Mycobacterium leprae*, leprosy, Hansen’s disease, laboratory diagnosis, biomarkers, PCR, SSS examination, and biopsies (protocol furnished as Figure 1). Out of 924, 715 full text articles were shortlisted. Twenty-seven of the 715 articles were included for the analysis based on these inclusion criteria: the study should have involved PCR as one of the tests for leprosy diagnosis or confirmation along with one other standard test such as SSS or biopsy and PCR tests conducted on (any) biological (clinical) samples. Exclusion criteria included articles published in a language other than English, articles which did not have free full text available, PCR used for purposes other than leprosy diagnosis, such as PCR used as a test for drug resistance, molecular typing, or where the PCR test was not used on clinical samples.

### 2.3. Data Extraction 

Each study was reviewed for the number of patients screened for leprosy diagnosis, mode of diagnosis including clinical examination and lab tests. Laboratory tests were further stratified into bacteriological examination by microscopy (AFB) and molecular tests (PCR) or histopathological examination (biopsy/HPE), clinical characteristics of the patients screened including PB/MB and nature of the clinical samples collected for lab diagnosis. Data on PCRs conducted on contacts/treated relapsed patients were excluded from the analysis. Percentage of positive results (sensitivity) for AFB microscopy and PCR were tabulated with reference to the clinical class of leprosy, AFB, and type of clinical sample used. Mean PCR positivity vis-à-vis genetic markers and the method of PCR used were also calculated.

### 2.4. Definitions of Some of the Data Terms Used in the Review

Report: Each individual paper included in the analysis is considered as one report;

Assay: PCR on each sample type and/or each gene marker from each report was considered as an independent assay; 

Gene Marker: each genetic marker used for specific amplification of *M. leprae* DNA from clinical specimens; 

PCR Method: laboratory technique of PCR used for amplifying the DNA targeting the *M. leprae* genetic markers.

### 2.5. Data Analysis

Data extraction has been carried out in such a way that PCR on each sample type and/or each gene marker was considered as an independent assay. Some of the papers reported PCR in one or more samples and/or one or more gene targets. Hence there were 38 assay resulting from the total of 27 papers that were reviewed. The matrix of published articles with types of lab tests conducted, types of samples used for testing, % PCR positivity, % AFB positivity on various samples, gene markers and/or PCR methods are tabulated for comparative analysis (Table 1). Mean and range are extracted for number of samples tested, percentage positivity of AFB, PCR for each clinical class, clinical specimen, PCR marker used, and PCR method used.

## 3. Results

### 3.1. Basic Clinical and Geographical Information

The review included published papers on PCR in new leprosy cases, along with clinical and conventional lab diagnostic tests. Out of 1700 papers screened, 27 papers qualified for the criteria of inclusion (flow chart, Figure 1). Out of the 27, nine were on leprosy patients from India, seven from Brazil, five from Bangkok, two from Philippines and one each from China, Ethiopia, Nepal, and Vietnam. Most of the reports included testing on clinically-diagnosed leprosy cases for confirming the laboratory test findings, limiting the scope of analysis for estimating only the sensitivity of the diagnostic tests. Most of those analyzed were reported based on either AFB microscopy on SSS or biopsies as the conventional lab standard test for comparing the PCR results.

### 3.2. Analysis

The average number of study subjects across the reports was 96 (range 20–439). Eighteen out of the 27 reports classified patients as PB and MB leprosy. Twenty-four (63%) out of 38 assays studied were less than 100 patients and 14 (37%) included more than 100 patients (Table 1). Seventeen out of 27 reports (62.9%) were based on a single PCR marker and nine (33%) used more than one marker; one (3.7%) used four markers. Three (8%) studies reported PCR results of a single marker on multiple samples (data not shown).

### 3.3. Clinical Classification vs. Sensitivity of AFB and PCR

Twenty-eight of the 38 of the total assays reported PCR on PB cases (six included data on AFB microscopy) (data not shown). Mean positivity for PCR was 48% (range 07–81) and AFB microscopy was 23% (range 1.7–35). Out of 38 assays, 26 reported PCR on MB leprosy cases and data on AFB was available for 17/26. Mean positivity for PCR was 77% (range 17–100) and AFB microscopy 59% (range 15–100) in reports on MB leprosy cases. Mean positivity for PCR was 44% (range 13–93) and AFB microscopy was 30% (range 10–86), if the total number of cases was not segregated as PB and MB (Table 2), indicating that PCR has only an incremental value over microscopy for bacteriological diagnosis of leprosy. Our observation on positivity of microscopy and PCR in PB leprosy cases reiterates the necessity to revise the current leprosy classification and treatment criteria. This also indicates that on an average 19% of cases were wrongly classified as PB (if only skin lesions were considered) and would have been missed treatment, had they not been tested with AFB and 10% more if PCR had not been used.

As expected, PCR turned out to be the most sensitive test for bacteriological confirmation on MB cases. Given the fact that MB leprosy lesions most frequently contain *M. leprae*, the mean PCR positivity in MB cases should have been more; however, the data suggests a lower average. This could have been due to technical bias due to errors in clinical sample collection and/or laboratory testing. These issues need to be addressed through development of robust clinical and lab protocols based on evidence from large multicentric studies through uniform study methodology. 

A few reports included PCR results stratified for Ridley-Jopling classes of leprosy. Azevedo et al., 2017 [13] reported PCR across the spectrum, with percentage of PCR positivity as TT-21/38 (55.2%); BT-20/21 (95.2%); BB-18/18 (100%); BL-12/12 (100%); LL-13/13 (100%). In another recent report Chaitanya et al., 2017, reported the results for a multiplex PCR as indeterminate 31/41 (75.6%) TT-03/03 (100%); BT- 40/42 (95.2%); BB-03/03 (100%); BL-58/59 (98%); LL-70/72 (97%). PCR showed a good additional value in diagnosing TT and indeterminate cases, which are traditionally known to be negative for AFB on microscopy. This indicates that PCR could be a better test in classifying bacillary-positive cases than smear and microscopy [21].

### 3.4. Clinical Specimens and PCR

De Wit et al. (1993) reported on the utility of PCR for detection of *M. leprae* in nasal swab specimens amplifying the 531-bp *pra* gene, demonstrating 79.6% positivity of PCR [22]. Kyeong-Han Yoon reported PCR on slit skin samples, which was subsequently reported by many others [23,24,25,26,27,28]. PCR on biopsies has been reported by Wichitwechkarn et al. (1995), with a mean PCR positivity of 66% and subsequently by many others. We found that biopsy was the most common specimen to have been tested for PCR of *M. leprae* [13,21,29,30,31,32,33,34,35,36,37] (Table 1).

Out of 38 protocols studied, the majority (20) were based on skin biopsy (53%) followed by SSS (13, 34%); the rest of the samples included nerve biopsy, blood, and urine. AFB positivity in SSS has been 36% (range 18–69) and skin biopsy 44% (range 10–85). Likewise, PCR positivity on slit skin and biopsy was 61% (range 18–93) and 70% (range 46–93), respectively (Table 2). Since the skin biopsy and SSS happen to be the most frequent clinical samples collected for leprosy diagnosis, we tried to analyze the usefulness of PCR in the two samples. PCR seems to be more sensitive than microscopy in both the type of samples (SSS and biopsies), although skin biopsies have demonstrated significantly higher sensitivity to both microscopy and PCR as compared to SSS (Table 2). The reason could be the presence of a lesser number of bacilli in SSS than those in biopsy. The data suggests that PCR on skin biopsy seems to be the most sensitive test for demonstrating *M. leprae* bacilli. Nevertheless, with 62% sensitivity, PCR on SSS seems to be a better test when compared to microscopy (36%). Given the inherent limitations of skin biopsy such as the invasive nature of collection and technical expertise needed for test reporting, PCR on SSS samples with an average sensitivity of 62% seems to be more practical for application at primary care levels.

Apart from these two conventional specimens, researchers also studied *M. leprae* PCR on other unconventional samples. Caleffi et al. (2012) used PCR, amplifying a 151-bp PCR fragment of the *M. leprae pra* gene in urine samples. Thirty four of the 73 (46.58%) leprosy patients studied were positive for PCR [38]. Tiwari et al. (2017) evaluated PCR in nerve biopsy specimens of 35 pure neuritic leprosy cases. AFB was positive in 13 (37.14%) cases and PCR positivity was observed in 22 (62.86%) cases [39]. A study involved 43 newly-diagnosed leprosy patients, where quantitative PCR was carried out on whole blood samples (PCR positivity—13.95%) in comparison with SSS of the ear lobe (microscopy—30.23%; PCR positivity—41.86%) [40]. It is interesting to note that even urine and blood samples could be used for PCR testing. These reports suggest that blood and urine, although less sensitive on PCR than SSS, may still be considered as potential specimens, owing to the convenience of sample collection at all levels of health care. Future research for generating more evidence on usefulness of PCR on urine and blood, both in terms of sensitivity and feasibility for a point of care test would be promising.

### 3.5. Gene Markers—PCR Sensitivity

Various published reports on PCR in leprosy diagnosis studied a spectrum of gene markers as PCR targets on various clinical samples by multiple laboratory methods. There were twelve different markers used for PCR testing (Table 1). RLEP and 16S rRNA are the most frequent markers used, with PCR sensitivity ranging between 57% and 80% for RLEP and from 13% to 82% for 16S rRNA. RLEP seems to be the most sensitive marker for detecting *M. leprae* DNA, although its variable performance across the assays needs to be further addressed (Table 3). The sensitivity of RLEP PCR varied between samples, between clinical settings, and also between studies of the same authors. Martinez et al. (2011) reported highest RLEP sensitivity (81%) as compared to other three PCR markers [34]. Maltempe et al. (2016) reported RLEP PCR on SSS to be equally sensitive as AFB smear microscopy (24%) [28], which is the lowest (data not shown). Yan et al. (2014) reported at least 72% of RLEP PCR positivity as compared to 35% on smear microscopy of paraffin-embedded biopsies among PB leprosy cases [36]. The observations paved the way for exploring more evidence on RLEP PCR, the most frequently used and promising marker, for reasons of its variable performance and opportunities for improving its effectiveness. It was also observed that multiplexing RLEP with other markers yielded better results (data not shown), indicating the necessity for undertaking more studies in this direction, with large sample numbers and uniform protocols simultaneously studied across multiple clinical sites. The data also suggested that the highest mean positivity with any of the markers so far reported seems to be only 71%, which needs to be improved if PCR is to be used as a robust test for diagnosing leprosy. Since DNA extraction is one of the critical steps in any PCR-based diagnosis, it is logical to look into impact of role of DNA extraction procedures on PCR outcomes. We observed that most of the assays reviewed were based on the standard DNA extraction protocols such as phenol:chloroform method. We did not find any specific protocol associated with a higher PCR sensitivity (data not shown). None of the assays had reported the use of DNA extraction controls, which could have validated the sample processing procedure.

There should be more research exploring better markers and suitable lab protocols for increasing the sensitivity of PCR for detecting *M. leprae*. Advancing scientific knowledge and an omics approach should throw some light on identifying such novel markers and developing them into robust PCR test modules for point-of-care diagnostic testing in leprosy.

### 3.6. Method of PCR vs. PCR Sensitivity

Out of 27 reports reviewed, 19 were based on conventional PCR, 6 were on quantitative real time PCR (qPCR), 4 on multiplex PCR and 2 were on reverse transcriptase PCR (RT-PCR) assays. Researchers have employed various PCR techniques for molecular diagnosis of *M. leprae* from SSS, skin biopsy, blood and urine samples. Conventional PCR targeting a single gene has been found to be the most frequently reported method. Several authors have used conventional PCR on slit skin, biopsy, urine, blood [27,37,38,39,40]. On the other hand, few studies have utilized multiplex PCR on slit skin and biopsies, amplifying more than one target sequence, at the same level of laboratory settings as conventional PCR [21,26,35,41]. There are also reports on methods such as quantitative PCR, RT-PCR and in situ PCR, which are based on advanced laboratory facility and studies have reported RT-PCR on skin biopsies, SSS and nasal biopsies [30,31,33,36,42]. Real-time quantitative (q-PCR) PCR technique was used by Martinez et al. (2011) and Gama et al. (2018) [34,40]. Yan et al. (2014) studied the nested PCR technique using two sets of primers to apply two different *M. leprae*-specific gene fragments from paraffin-embedded skin biopsy specimens [36]. Dayal et al. (2005) and Kamal et al. (2010) reported in situ PCR [24,43].

From the above analyzed assay, the highest percentage of PCR sensitivity was observed using multiplex PCR technique (82%) followed by RT-PCR (78%) and conventional PCR (63%). These two techniques seem to be very useful PCR techniques in the rapid diagnosis of *M. leprae*. This observation indicates that even a simple PCR but with more than one marker and appropriate technical protocol could do better in picking up *M. leprae* DNA from clinical samples. This in turn indicates the usefulness of simple PCR-based tests for bacteriological diagnosis of leprosy even with a modestly-equipped laboratory facility, although RT-PCR carries a technical advantage over simple PCR by providing quantitative estimate. 

The review analysis found that no individual *M. leprae* gene marker has been associated with higher sensitivity. However, using more than one marker in a multiplex format of conventional PCR seem to have yielded significantly higher mean positivity and hence could be a better choice (Table 3). Almost all studies reported in this review have studied techniques based on traditional laboratory-based PCRs, since the evidence gathered through this review suggests that it is more important to have appropriate protocols and robust markers for PCR sensitivity than sophisticated technology. Recent advancements in molecular methods has enhanced the capability of detection and characterization of infectious diseases. PCR is one of the techniques of molecular diagnosis that has great benefits and enhances advancements in rapid diagnosis. There are many simple point of care (POC) PCRs such as the LAMP test and Xpert TB available for diseases like TB, HIV, and malaria etc. [47,48,49,50,51,52]. Developing one or more of the existing markers into such POC platforms should be the goal of future research in molecular diagnosis of leprosy. Developing such point-of-care, easy-to-use PCR modules could have potential application at all healthcare levels. This addresses the issue of access to healthcare for persons affected by leprosy or those at risk of developing leprosy. Increasing access to healthcare leads to early detection of leprosy and prevention of consequences—the most important aspects of leprosy control. Hence it is envisaged that further research on PCR in leprosy include investigating various markers for the usefulness of point-of-care testing.

## 4. Conclusions

The evidence from this review analysis suggests that PCR on a skin biopsy is the ideal diagnostic test. Nevertheless, PCR on SSS samples also seems to be useful with its practical value for application at primary healthcare levels. Future research for better evidence on the usefulness of PCR on other samples such as urine and blood are recommended to avoid collecting slit skin/biopsy samples, which are relatively more invasive. Our observation on positivity of AFB microscopy and PCR in PB leprosy cases reiterates the necessity to revise the current leprosy classification and treatment criteria based merely on the number of skin lesions. PCR could be a better test in classifying bacillary positive cases than smear microscopy. Having said that, our review findings indicate the necessity for improving the sensitivity of PCR and further research on specificity is required in ruling out other clinical conditions that may mimic leprosy. Development of robust clinical and lab protocols based on evidence from large multicenter studies through uniform study methodology might be of great help in addressing the sensitivity and specificity issues. We found that no individual *M. leprae* gene marker has been associated with higher PCR sensitivity, indicating the need for more evidence for robust markers. However, using more than one marker in a multiplex format of conventional PCR seems to have yielded significantly higher PCR positivity and hence could be a better choice. RLEP, although the most frequent marker used, showed variable performance across the clinical sites, and samples are a matter of concern. Combining it with other markers in a multiplex PCR format might work. Multiplexing more than one target *M. leprae* gene might also help to address this issue. Undertaking more studies of this nature, with large sample numbers and uniform protocols simultaneously studied across multiple clinical sites would be useful. In addition, there should be more research exploring better markers for increasing sensitivity of PCR for detecting *M. leprae*. Advancing scientific knowledge and omics approach should throw some light on the identification of such novel markers and developing them into robust PCR test modules for point-of-care diagnostic testing in leprosy.

### Limitations of This Systematic Review Analysis

The present review has been mainly based on the reports available on the PubMed database and did not include any other databases due to the time and resource constraints. This might have led to a selection bias of papers published only on PubMed. We did not gather any other types of information sources such as unpublished data, data from ongoing studies from various researchers, which could perhaps have made our observations stronger. The data from records mainly comprising clinically-diagnosed leprosy cases, except one record, meant that we were unable to estimate test specificity. Analysis included data only on new patients. However, the authors would like to address the other two important applications of PCR, namely for contact screening and treatment monitoring through separate independent reviews. We limited the analysis to systematic review only and did not attempt meta-analysis due to heterogeneity of the data. This review could not elicit any significant evidence on usefulness of any specific marker for PCR, due to either limited number of studies or limited number of samples. Further studies with large sample sizes with existing or new markers are warranted to enhance evidence on this particular aspect.

## Figures and Tables

**Table 1:** Data showing different studies involving conventional and molecular diagnosis of leprosy using different samples.

Sl. No.	Type of Sample	PCR Type	Marker/Gene	No. of Patients Studied	Smear Microscopy			PCR			*p* Value	First Author	Study Location/Country	Reference No.
					No. Tested	No. Positive	%	No. Tested	No. Positive	%				
1	Skin biopsy	Conventional PCR	RLEP	102	102	63	61.76	102	59	57.84	NA	Michelle de Campos Soriani Azevedo	Brazil	[13]
2	Skin biopsy	Multiplex PCR	RLEP	220	220	122	55.45	220	164	74.55	*p* < 0.05	V Sundeep Chaitanya	India	[21]
3	Skin biopsy	Multiplex PCR	M- PCR	220	220	122	55.45	220	205	93.18	NA	V Sundeep Chaitanya	India	[21]
4	Nasal swabs	Conventional PCR	531 bp fragment	103	0	0	0	103	82	79.61	NA	Madeleine Y. L. de Wit	Philippines	[22]
5	SSS	Conventional PCR	372 bp fragment	102	102	62	60.78	102	95	93.14	NA	Kyeong-Han Yoon	Philippines	[23]
6	Skin biopsy	Conventional PCR	372 bp	102	102	87	85.29	102	95	93.14	NA	Kyeong-Han Yoon	Philippines	[23]
7	SSS	in situ PCR	530 bp fragment	25	25	5	20	25	18	72	*p* = 0.01	R Kamal	India	[24]
8	SSS	Conventional PCR	RLEP	73	73	17	23.29	73	56	76.71	*p* < 0.001	R Kamal	India	[25]
9	SSS	Multiplex PCR	372&201 bp	439	439	223	50.8	439	371	84.51	NA	Surajita Banerjee	India	[26]
10	SSS	Conventional PCR	RLEP	50	50	9	18	50	36	72	NA	Shraddha Siwakoti	Nepal	[27]
11	SSS	Conventional PCR	PCR-LP	91	91	21	23.08	91	22	24.18	NA	Flaviane Granero Maltempe	Brazil	[28]
12	SSS	Conventional PCR	PCR-P	91	91	21	23.08	91	17	18.68	*p* > 0.05	Flaviane Granero Maltempe	Brazil	[28]
13	SSS	Conventional PCR	*pra* gene	53	0	0	0	53	17	32.08	NA	Jesdawan Wichitwechkaran	Bangkok	[29]
14	Skin biopsy	Conventional PCR	*pra* gene	53	0	0	0	53	35	66.04	NA	Jesdawan Wichitwechkaran	Bangkok	[29]
15	Skin biopsy	RT-PCR	16S rRNA	50	50	33	66	50	41	82	NA	Mekonnen Kurabachew	Ethiopia	[30]
16	Nasal mucosal biopsies	RT-PCR	16S rRNA	60	60	24	40	60	47	78.33	NA	Benjawan Phetsuksiri	Bangkok	[31]
17	Skin biopsy	Conventional PCR	RLEP	110	110	43	39.09	110	81	73.64	NA	Isabela Maria Bernardes Goulart	Brazil	[32]
18	Skin biopsy	Conventional PCR	372 bp	110	110	43	39.09	110	58	52.73	NA	Isabela Maria Bernardes Goulart	Brazil	[32]
19	Skin biopsy	qPCR	16S rRNA	69	69	0	0	69	53	76.81	NA	Pham Dang Bang	Vietnam	[33]
20	Skin biopsy	qPCR	RLEP	47	0	0	0	47	38	80.85	NA	Alejandra Nóbrega Martinez	Brazil	[34]
21	Skin biopsy	qPCR	16S rRNA	47	0	0	0	47	24	51.06	NA	Alejandra Nóbrega Martinez	Brazil	[34]
22	Skin biopsy	qPCR	*sodA*	47	0	0	0	47	22	46.81	NA	Alejandra Nóbrega Martinez	Brazil	[34]
23	Skin biopsy	qPCR	85B	47	0	0	0	47	26	55.32	NA	Alejandra Nóbrega Martinez	Brazil	[34]
24	Skin biopsy	Multiplex PCR	372bp &201 bp	165	165	84	50.91	165	111	67.27	NA	Abu Hena Hasanoor Reja	India	[35]
25	Skin biopsy	qPCR	RLEP & 372 bp fragment	51	51	18	35.29	51	38	74.51	*p >* 0.05	Wen Yan	China	[36]
26	Skin biopsy	Nested PCR	RLEP &372 bp fragment	51	51	18	35.29	51	37	72.55	NA	Wen Yan	China	[36]
27	Skin biopsy	Conventional PCR	530 bp fragment	55	55	9	16.36	55	40	72.73	NA	Mohammad Shah Alam	Bangladesh	[37]
28	Urine	Conventional PCR	*pra* gene	73	73	0	0	73	34	46.58	*p* > 0.05	K.R. Caleffi	Brazil	[38]
29	Nerve biopsy	Conventional PCR	375 bp fragment	35	35	13	37.14	35	22	62.86	NA	Vandana Tiwari	India	[39]
30	SSS	qPCR	16S rRNA	43	43	13	30.23	43	18	41.86	NA	Rafael Silva Gama	Brazil	[40]
31	blood	qPCR	16S rRNA	43	0	0	0	43	6	13.95	NA	Rafael Silva Gama	Brazil	[40]
32	SSS	Multiplex PCR	372 bp fragment	164	164	65	39.63	164	135	82.32	*p* < 0.0001	Surajita Banerjee	India	[41]
33	SSS	qPCR	16S rRNA	66	66	36	54.55	66	52	78.79	NA	Janisara Rudeeaneksin	Bangkok	[42]
34	Skin biopsy	in situ PCR	530 bp fragment	20	20	2	10	20	12	60	NA	R. Dayal	India	[43]
35	SSS	Conventional PCR	*pra* gene	122	122	49	40.16	122	86	70.49	*p* < 0.001	Kowit Kampirapap	Bangkok	[44]
36	Skin biopsy	Conventional PCR	RLEP	180	180	122	67.78	180	114	63.33	*p* < 0.0001	V Sundeep Chaitanya	India	[45]
37	Skin biopsy	Conventional PCR	ML1545	180	180	122	67.78	180	164	91.11	NA	V Sundeep Chaitanya	India	[45]
38	SSS	Conventional PCR	372 bp fragment	52	52	36	69.23	52	36	69.23	NA	Lucas Gomes Patrocínio	Brazil	[46]

**Table 2:** Details of assay—clinical classification, clinical sample vs. smear microcopy and polymerase chain reaction (PCR) positivity.

Classification	No. of Assay (Reports) Studied	Average Number of Patients/Samples Tested (Range)	No. of Assay Reported the AFB Microscopy	%AFB Positivity Mean (Range)	No. of Assay Reported PCR Tests	%PCR Positivity
Bacillary Load						
Paucibacillary	28	37.07 (7–234)	6	25.18 (1.75–35.29)	28	48.63 (7.69–81)
Multibacillary	27	61.40 (12–205)	17	62.25 (15.38–100)	27	79.65 (17.39–100)
Clinical samples						
Slit skin samples	14	101 (25–439)	12	37.73 (18–69.23)	14	60.71 (18.68–93.14)
Skin biopsy	20	96.3 (20–220)	14	48.96 (10–85.29)	20	70.27 (46.81–93.18)

**Table 3:** PCR markers and methods vs. sensitivity.

	Number of Assays Studied (*n* = 38)	Highest Positivity (%PCR)	Lowest Positivity (%PCR)
**Gene markers**			
RLEP	9	80.85	57.84
16S rRNA	10	82	13.95
**Method of PCR**			
Conventional	19	93.14	18.68
Multiplex	6	93.18	67.27
Q-PCR	6	80.85	13.95
RT-PCR	5	74.5	82

## References

[1] Noordeen S.K., Hastings R.C. (1985). The epidemiology of leprosy. Leprosy.

[2] Dockrell H.M., Bryceson A.D.M., Pfaltzgraff R.E. (1990). Leprosy.

[3] Lockwood D.N.J. (2005). Leprosy. Medicine.

[4] Van Brakel W.H., Sihombing B., Djarir H., Beise K., Kusumawardhani L., Yulihane R., Kurniasari I., Kasim M., Kesumaningsih K.I., Wilder-Smith A. (2012). Disability in people affected by leprosy: The role of impairment, activity, social participation, stigma and discrimination. Glob. Health Action..

[5] World Health Organization (2002). Global Target Attained, Remaining Endemic Countries Pose Greatest Challenge.

[6] World Health Organization (2016). The Global Leprosy Strategy 2016–2020: Accelerating towards a Leprosy-Free World.

[7] World Health Organization (2017). Global leprosy update, 2016: Accelerating reduction of disease burden. Wkly. Epidemiol. Rec..

[8] Sermrittirong S., van Brakel W.H. (2014). Stigma in leprosy: Concepts, causes and determinants. Lepr. Rev..

[9] Duthie M.S., Truman R.W., Goto W., O’Donnell J., Hay M.N., Spencer J.S., Carter D., Reed S.G. (2011). Insight toward early diagnosis of leprosy through analysis of the developing antibody responses of *Mycobacterium leprae*-infected armadillos. Clin. Vaccine Immunol..

[10] Veena S., Kumar P., Shashikala P., Gurubasavaraj H., Chandrasekhar H.R. (2011). Significance of histopathology in leprosy patients with 1–5 skin lesions with relevance to therapy. J. Lab. Physicians.

[11] Lockwood D.N., Nicholls P., Smith W.C., Das L., Barkataki P., van Brakel W., Suneetha S. (2012). Comparing the clinical and histological diagnosis of leprosy and leprosy reactions in the INFIR cohort of Indian patients with multibacillary leprosy. PLoS Negl. Trop. Dis..

[12] Fischer M. (2017). Leprosy—An overview of clinical features, diagnosis, and treatment. J. Dtsch. Dermatol. Ges..

[13] Lastória J.C., Abreu M.A. (2014). Leprosy: A review of laboratory and therapeutic aspects—Part 2. An. Bras. Dermatol..

[14] Azevedo M.C., Ramuno N.M., Fachin L.R., Tassa M., Rosa P.S., Belone A.F., Diorio S.M., Soares C.T., Garlet G.P., Trombone A.P. (2017). qPCR detection of *Mycobacterium leprae* in biopsies and slit skin smear of different leprosy clinical forms. Braz. J. Infect. Dis..

[15] Speers D.J. (2006). Clinical applications of molecular biology for infectious diseases. Clin. Biochem. Rev..

[16] Niemz A., Boyle D.S. (2012). Nucleic acid testing for tuberculosis at the point-of-care in high-burden countries. Expert Rev. Mol. Diagn..

[17] Martinez A.N., Talhari C., Moraes M.O., Talhari S. (2014). PCR-based techniques for leprosy diagnosis: From the laboratory to the clinic. PLoS Negl. Trop. Dis..

[18] Male M.M., Rao B.G., Chokkakula S., Kasetty S., Rao P.V.R., Jonnalagada S., Reddy A.M., Srikantam A. (2016). Molecular screening for primary drug resistance in *M. leprae* from newly diagnosed leprosy cases from India. Lepr. Rev..

[19] Moher D., Liberati A., Tetzlaff J., Altman D.G., Group P. (2009). Preferred reporting items for systematic reviews and meta-analyses: The PRISMA statement. Ann. Intern. Med..

[20] Canese K., Weis S. (2013). PubMed: The Bibliographic Database. The NCBI Handbook [Internet].

[21] Chaitanya V.S., Cuello L., Das M., Sudharsan A., Ganesan P., Kanmani K., Rajan L., Ebenezer M. (2017). Analysis of a novel multiplex polymerase chain reaction assay as a sensitive tool for the diagnosis of indeterminate and tuberculoid forms of leprosy. Int. J. Mycobacteriol..

[22] De Wit M.Y., Douglas J.T., McFadden J., Klatser P.R. (1993). Polymerase chain reaction for detection of *Mycobacterium leprae* in nasal swab specimens. J. Clin. Microbiol..

[23] Yoon K.H., Cho S.N., Lee M.K., Abalos R.M., Cellona R.V., Fajardo T.T., Guido L.S., Dela Cruz E.C., Walsh G.P., Kim J.D. (1993). Evaluation of polymerase chain reaction amplification of mycobacterium leprae-specific repetitive sequence in biopsy specimens from leprosy patients. J. Clin. Microbiol..

[24] Kamal R., Natrajan M., Katoch K., Katoch V.M. (2010). Evaluation of diagnostic role of in situ PCR on slit-skin smears in pediatric leprosy. Indian J. Lepr..

[25] Kamal R., Dayal R., Gaidhankar K., Biswas S., Gupta S.B., Kumar N., Kumar R., Pengoria R., Chauhan DS., Katoch K. (2016). RLEP PCR as a definitive diagnostic test for leprosy from skin smear samples in childhood and adolescent leprosy. Indian J. Lepr..

[26] Banerjee S., Sarkar K., Gupta S., Mahapatra P.S., Gupta S., Guha S., Bandhopadhayay D., Ghosal C., Paine S.K., Dutta R.N. (2010). Multiplex PCR technique could be an alternative approach for early detection of leprosy among close contacts—A pilot study from India. BMC Infect. Dis..

[27] Siwakoti S., Rai K., Bhattarai N.R., Agarwal S., Khanal B. (2016). Evaluation of polymerase chain reaction (PCR) with slit skin smear examination (SSS) to confirm clinical diagnosis of leprosy in eastern Nepal. PLoS Negl. Trop. Dis..

[28] Maltempe F.G., Baldin V.P., Lopes M.A., Siqueira V.L.D., de Lima Scodro R.B., Cardoso R.F., Caleffi-Ferracioli K.R. (2016). Critical analysis: Use of polymerase chain reaction to diagnose leprosy. Braz. J. Pharm. Sci..

[29] Wichitwechkarn J., Karnjan S., Shuntawuttisettee S., Sornprasit C., Kampirapap K., Peerapakorn S. (1995). Detection of *Mycobacterium leprae* infection by PCR. J. Clin. Microbiol..

[30] Kurabachew M., Wondimu A., Ryon J.J. (1998). Reverse transcription-PCR detection of *Mycobacterium leprae* in clinical specimens. J. Clin. Microbiol..

[31] Phetsuksiri B., Rudeeaneksin J., Supapkul P., Wachapong S., Mahotarn K., Brennan P.J. (2006). A simplified reverse transcriptase PCR for rapid detection of *Mycobacterium leprae* in skin specimens. FEMS Immunol. Med. Microbiol..

[32] Goulart I.M., Cardoso A.M., Santos M.S., Goncalves M.A., Pereira J.E., Goulart L.R. (2007). Detection of *Mycobacterium leprae* DNA in skin lesions of leprosy patients by PCR may be affected by amplicon size. Arch. Dermatol. Res..

[33] Bang P.D., Suzuki K., Phuong le T., Chu T.M., Ishii N., Khang T.H. (2009). Evaluation of polymerase chain reaction-based detection of *Mycobacterium leprae* for the diagnosis of leprosy. J. Dermatol..

[34] Martinez A.N., Ribeiro-Alves M., Sarno E.N., Moraes M.O. (2011). Evaluation of qPCR-based assays for leprosy diagnosis directly in clinical specimens. PLoS Negl. Trop. Dis..

[35] Reja A.H., Biswas N., Biswas S., Dasgupta S., Chowdhury I.H., Banerjee S., Chakraborty T., Dutta P.K., Bhattacharya B. (2013). Fite-Faraco staining in combination with multiplex polymerase chain reaction: A new approach to leprosy diagnosis. Indian J. Dermatol. Venereol. Leprol..

[36] Yan W., Xing Y., Yuan L.C., De Yang R., Tan F.Y., Zhang Y., Li H.Y. (2014). Application of RLEP real-time PCR for detection of *M. leprae* DNA in paraffin-embedded skin biopsy specimens for diagnosis of paucibacillary leprosy. Am. J. Trop. Med. Hyg..

[37] Alam M.S., Shamsuzzaman S.M., Mamun K.Z. (2017). Demography, clinical presentation and laboratory diagnosis of leprosy by microscopy, histopathology and PCR from Dhaka city in Bangladesh. Lepr. Rev..

[38] Caleffi K.R., Hirata R.D., Hirata M.H., Caleffi E.R., Siqueira V.L., Cardoso R.F. (2012). Use of the polymerase chain reaction to detect *Mycobacterium leprae* in urine. Braz. J. Med. Biol. Res..

[39] Tiwari V., Malhotra K., Khan K., Maurya P.K., Singh A.K., Thacker A.K., Husain N., Kulshreshtha D. (2017). Evaluation of polymerase chain reaction in nerve biopsy specimens of patients with Hansen’s disease. J. Neurol. Sci..

[40] Gama R.S., Gomides T.A.R., Gama C.F.M., Moreira S.J.M., de Neves Manta F.S., de Oliveira L.B.P., Marcal P.H.F., Sarno E.N., Moraes M.O., Garcia R.M.G. (2018). High frequency of *M. leprae* DNA detection in asymptomatic household contacts. BMC Infect. Dis..

[41] Banerjee S., Biswas N., Kanti Das N., Sil A., Ghosh P., Hasanoor Raja A.H., Dasgupta S., Kanti Datta P., Bhattacharya B. (2011). Diagnosing leprosy: Revisiting the role of the slit-skin smear with critical analysis of the applicability of polymerase chain reaction in diagnosis. Int. J. Dermatol..

[42] Rudeeaneksin J., Srisungngam S., Sawanpanyalert P., Sittiwakin T., Likanonsakul S., Pasadorn S., Palittapongarnpim P., Brennan P.J., Phetsuksiri B. (2008). LightCycler real-time PCR for rapid detection and quantitation of *Mycobacterium leprae* in skin specimens. FEMS Immunol. Med. Microbiol..

[43] Dayal R., Singh S.P., Mathur P.P., Katoch V.M., Katoch K., Natrajan M. (2005). Diagnostic value of in situ polymerase chain reaction in leprosy. Indian J. Pediatr..

[44] Kampirapap K., Singtham N., Klatser P.R., Wiriyawipart S. (1998). DNA amplification for detection of leprosy and assessment of efficacy of leprosy chemotherapy. Int. J. Lepr. Other Mycobact. Dis..

[45] Sundeep Chaitanya V., Das M., Eisenbach T.L., Amoako A., Rajan L., Horo I., Ebenezer M. (2016). *Mycobacterium leprae* specific genomic target in the promoter region of probable 4-alpha-glucanotransferase (ML1545) gene with potential sensitivity for polymerase chain reaction based diagnosis of leprosy. Int. J. Mycobacteriol..

[46] Patrocínio L.G., Goulart I.M., Goulart L.R., Patrocinio J.A., Ferreira F.R., Fleury R.N. (2005). Detection of *Mycobacterium leprae* in nasal mucosa biopsies by the polymerase chain reaction. FEMS Immunol. Med. Microbiol..

[47] Boehme C.C., Nabeta P., Henostroza G., Raqib R., Rahim Z., Gerhardt M., Sanga E., Hoelscher M., Notomi T., Hase T. (2007). Operational feasibility of using loop-mediated isothermal amplification for diagnosis of pulmonary tuberculosis in microscopy centers of developing countries. J. Clin. Microbiol..

[48] Boehme C.C., Nabeta P., Hillemann D., Nicol M.P., Shenai S., Krapp F., Allen J., Tahirli R., Blakemore R., Rustomjee R. (2010). Rapid molecular detection of tuberculosis and rifampin resistance. N. Engl. J. Med..

[49] Mori Y., Notomi T. (2009). Loop-mediated isothermal amplification (LAMP): A rapid, accurate, and cost-effective diagnostic method for infectious diseases. J. Infect. Chemother..

[50] Lawn S.D., Nicol M.P. (2011). Xpert^®^ MTB/RIF assay: Development, evaluation and implementation of a new rapid molecular diagnostic for tuberculosis and rifampicin resistance. Future Microbiol..

[51] Arora D.R., Maheshwari M., Arora B. (2013). Rapid point-of-care testing for detection of HIV and clinical monitoring. ISRN AIDS.

[52] Kim S., Nhem S., Dourng D., Ménard D. (2015). Malaria rapid diagnostic test as point-of-care test: Study protocol for evaluating the VIKIA^®^ Malaria Ag Pf/Pan. Malar. J..

